# miR-22-3p Negatively Affects Tumor Progression in T-Cell Acute Lymphoblastic Leukemia

**DOI:** 10.3390/cells9071726

**Published:** 2020-07-18

**Authors:** Valentina Saccomani, Angela Grassi, Erich Piovan, Deborah Bongiovanni, Ludovica Di Martino, Sonia Minuzzo, Valeria Tosello, Paola Zanovello

**Affiliations:** 1 Department of Surgery, Oncology and Gastroenterology, Immunology & Oncology Section, University of Padova, 35128 Padua, Italy; valentina.saccomani88@gmail.com (V.S.); erich.piovan@unipd.it (E.P.); deborah.bongiovanni@studenti.unipd.it (D.B.); ludovica.dimartino@studenti.unipd.it (L.D.M.); soniaanna.minuzzo@unipd.it (S.M.); 2 Immunology and Molecular Oncology Unit, Veneto Institute of Oncology IOV—IRCCS, 35128 Padua, Italy; angela.grassi@unipd.it

**Keywords:** miR-22-3p, T-ALL, NOTCH1

## Abstract

T-cell acute lymphoblastic leukemia (T-ALL) is a rare, aggressive disease arising from T-cell precursors. NOTCH1 plays an important role both in T-cell development and leukemia progression, and more than 60% of human T-ALLs harbor mutations in components of the NOTCH1 signaling pathway, leading to deregulated cell growth and contributing to cell transformation. Besides multiple NOTCH1 target genes, microRNAs have also been shown to regulate T-ALL initiation and progression. Using an established mouse model of T-ALL induced by NOTCH1 activation, we identified several microRNAs downstream of NOTCH1 activation. In particular, we found that NOTCH1 inhibition can induce miR-22-3p in NOTCH1-dependent tumors and that this regulation is also conserved in human samples. Importantly, miR-22-3p overexpression in T-ALL cells can inhibit colony formation in vitro and leukemia progression in vivo. In addition, miR-22-3p was found to be downregulated in T-ALL specimens, both T-ALL cell lines and primary samples, relative to immature T-cells. Our results suggest that miR-22-3p is a functionally relevant microRNA in T-ALL whose modulation can be exploited for therapeutic purposes to inhibit T-ALL progression.

## 1. Introduction

T-cell acute lymphoblastic leukemia (T-ALL) results from the malignant transformation of T-cell progenitors that cause diffuse infiltration of the bone marrow by immature T-cell lymphoblasts.

High-dose multi-agent chemotherapy regimens can cure 80% of pediatric patients, but at least 20% of pediatric and 40% of adult T-ALL patients eventually relapse, leading to poor prognosis. Unfortunately, the specific mechanisms mediating escape from therapy, disease progression, and leukemia relapse remain largely unknown [1].

T-cell transformation is characterized by an accumulation of genomic alterations that generate oncogenes and inactivate tumor suppressor genes, contributing to uncontrolled cell proliferation and cell cycle progression, differentiation arrest, and abnormal cellular metabolism. A central role in T-cell transformation is played by NOTCH1, which is a key transmembrane transcription factor involved in T-cell fate specification and development and is activated in over 60% of cases [2]. Upon interaction with its ligands, NOTCH1 undergoes a conformational change that leads to two sequential proteolytic cleavages, first by an ADAM metalloprotease and subsequently by the γ-secretase complex. This final cleavage releases the intracellular domains of NOTCH1 (ICN1) from the membrane, allowing its translocation to the nucleus, where it activates gene expression via association with CBF1, Suppressor of Hairless, and Lag-1 (CSL). Patients carrying mutations in the *NOTCH1* gene constitutively express high levels of ICN1. The NOTCH1 oncogenic program can be therapeutically targeted by small-molecule γ-secretase inhibitors (GSIs), which effectively block NOTCH1 activation via the inhibition of a critical intramembrane proteolytic cleavage that is required for NOTCH1 signaling, making NOTCH1 signaling an important therapeutic target in T-ALL. During T-cell transformation, high levels of activated Notch1 in murine T-cell progenitor models impair T-cell maturation, leading to the accumulation of CD4pos/CD8pos cells, promote thymic-independent T-cell development, and ultimately lead to T-cell leukemia [3]. In fact, Notch directly regulates pre-T-cell antigen receptor α (*Ptcra*), interleukin 7 receptor (*Il7r*), and *Igf1r* genes [4,5,6]. Moreover, NOTCH1 directly upregulates genes that control anabolic metabolism, including those involved in biosynthesis, protein translation, and nucleotide and amino acid metabolism, mainly through direct transcriptional regulation of the *MYC* oncogene [7,8].

Non-coding RNAs (ncRNAs) have emerged as crucial players in post-transcriptional gene regulation. Among the ncRNAs are microRNAs (miRNAs), which control target mRNAs through degradation or translational repression and are reported to regulate different biological processes, including development, differentiation, and cancer [9,10]. Recently, miRNAs that may play critical roles in the NOTCH signaling pathway have been identified using different approaches, from genetic screens to miRNA profiling, by comparing normal T-cell subsets with NOTCH1-driven leukemia [11,12,13,14]. However, little is currently known about miRNAs that are regulated in therapeutic contexts, such as NOTCH1 blockage with gamma-secretase inhibitors. Using T-ALL cell lines and inhibiting NOTCH1 in vitro, Guascott et al. identified only a few miRNAs, mainly because of the heterogeneity among the analyzed cell lines [15]. In our study, we took advantage of a mouse model of NOTCH1-induced T-cell leukemia that is strictly dependent on this oncogene and performed in vivo NOTCH1 inhibition using a gamma-secretase inhibitor. This analysis allowed us to identify novel miRNAs that may act in concert with NOTCH1 to play a role in in vivo T-ALL progression. We focused our research on miR-22-3p, one of the most significantly modulated miRNAs whose function in T-ALL is still ill defined.

## 2. Materials and Methods

### 2.1. Mouse Models of NOTCH1-Induced T-ALL

As previously reported [3,16], retrovirus-mediated overexpression of activated NOTCH1 alleles in hematopoietic lineage-negative progenitors induces primarily ectopic T-cell development and secondary T-cell leukemia. Different *NOTCH1* alleles can recapitulate T-cell leukemia in the mouse: the HD-ΔPEST allele contains a mutation in the HD (heterodimerization) domain (L1601P) and a deletion in the PEST (proline (P), glutamic acid (E), serine (S), and threonine (T)) domain (ΔPEST) that closely resembles a human *NOTCH1* mutation, and the ΔE allele presents a truncated NOTCH1 that resembles NOTCH1 translocation found in about 1–3% of patients. Both alleles are sensitive to gamma-secretase inhibitors.

We generated NOTCH1-induced tumors using both HD-ΔPEST and ΔE alleles, as previously described [16,17]. Tumor-bearing mice were euthanized, and primary tumor cells were extracted from their spleens. These tumor cells were then re-injected into sub-lethally irradiated mice (4 Gy) to generate secondary NOTCH1-induced T-ALL tumors. When these mice showed signs of leukemia development, groups of mice were randomized and injected intraperitoneally (i.p.) with three doses of dibenzazepine (DBZ) (5 mg/kg), which is a potent GSI, or Dimethyl sulfoxide (DMSO, vehicle) at 8 h intervals. Each experimental group consisted of at least three animals. After this treatment, mice were sacrificed, and T-leukemia cells were isolated from infiltrated spleens to perform molecular analyses. Procedures involving animals and their care conformed with institutional guidelines that comply with national and international laws and policies (EEC Council Directive 86/609, OJ L 358, 12 206 December 1987). All mice were monitored daily, and animals showing overt signs of disease or excessive weight loss were euthanized following Institutional Animal Care and Use Committee guidelines. These experiments were authorized by the Italian Ministry of Health (authorization n° 136/2014 released the 28th of March 2014).

### 2.2. Microarray Experiments and Bioinformatic Analyses

Total RNA from the spleens of NOTCH1 (HD-∆PEST)-induced T-leukemia-bearing mice treated with DBZ (*n* = 3) or DMSO (*n* = 3) was extracted using TRIzol according to the manufacturer’s instructions. RNA quality and purity were assessed using an Agilent Bioanalyzer 2100 (Agilent Technologies, Waldbronn, Germany) at CRIBI Biotechnology Center (University of Padova). Only RNA samples that passed quality control were used to perform gene expression profiling, which was carried out using SurePrint G3 Mouse GE 8 × 60 K (Agilent Technologies). MiRNA expression profiling was performed using mouse miRNA 8 × 60 K release 19.0 (Agilent Technologies). Microarray slides were scanned using an Agilent microarray scanner system (G2505C for gene and G2565CA for miRNA expression) following the manufacturer’s instructions. Feature Extraction software version 10.7.3.1 (Agilent Technologies) (including the optimal grid file, 028005_D_F_20100804 for gene and 046065_D_F_20121223 for miRNA expression microarrays) was used with the recommended settings to quantify hybridization signals and produce quality control reports and raw data for bioinformatic analyses.

Bioinformatic analysis of gene expression microarray data was performed in the R/Bioconductor statistical environment using the limma package. Data were preprocessed using the ‘Normexp’ background correction method with an offset of 16, and normalization between arrays was executed with the quantile method. Differential expression analysis was performed with a linear model, and gene-wide moderated t-statistics were obtained by empirical Bayes shrinkage. The Benjamini–Hochberg method was used to correct for multiple testing using a strict false discovery rate (FDR) cut-off of 0.01. Gene set enrichment analysis (GSEA) was performed to evaluate the functional significance of curated gene sets. Genes were ranked by decreasing moderated t-statistics, and GSEAPreranked was run with default parameters. Gene sets in the H collection of the Molecular Signatures Database (MSigDB) v7.0, consisting of 50 hallmark gene sets, were tested for significance.

For miRNA analysis, raw data were loaded and preprocessed in the R statistical environment using the AgiMicroRna Bioconductor library. Preprocessing was performed using the robust multi-array average (RMA) algorithm to yield a summary measure of the miRNA expression using a linear model that accounts for the probe affinity effect. As suggested by López-Romero [18,19], background correction was omitted as it can increase the false-positive detection of fold changes in low-expressed miRNAs. Differential expression analysis was performed using the limma Bioconductor package, followed by multiple testing correction using the Benjamini–Hochberg method. MiRNAs with absolute fold change (FC) > 1.3 and FDR < 0.05 were considered differentially expressed.

Transcriptome profiling of the MOLT4 cell line overexpressing miR-22-3p (*n* = 3) or the empty vector (*n* = 3) was carried out using the human Clariom™ S Assay (ThermoFisher Scientific, Waltham, MA, USA). Microarray data were analyzed in the R/Bioconductor statistical environment using the RMA algorithm for normalization and the limma package for differential expression analysis. Gene set enrichment analysis was performed to test the significance of gene ontology (GO) gene sets in the MSigDB v7.0 C5 collection corresponding to the molecular function (MF) and biological process (BP) ontologies.

Raw microarray data, together with the description of experiments, protocols and results of differential expression analysis, have been deposited in the ArrayExpress database (www.ebi.ac.uk/arrayexpress) under accession numbers E-MTAB-9278 (mouse miRNA arrays), E-MTAB-9279 (mouse gene expression arrays) and E-MTAB-9280 (human gene expression arrays).

### 2.3. In Vivo Experiments with Xenografts

Primary T-ALL samples were obtained at the time of diagnosis with informed consent from the Oncohematology Laboratory, Department of Woman and Child Health, University of Padova, according to the guidelines of the local ethics committee. Patient-derived xenografts (PDX) were previously established and characterized for *NOTCH1* and *FBXW7* mutations by Dr. Stefano Indraccolo at the Veneto Institute of Oncology, IRCCS, Padua [20,21]. PDX cells were thawed, and 10 × 10^6^ viable T-ALL cells (in 400 µL of PBS) were injected intravenously (i.v.) in 6–8-week-old NOD.Cg-*Prkdc^scid^ Il2rg^tm1Wjl^*/SzJ (NSG) immunodeficient mice. The degree of T-ALL engraftment in mice was monitored by periodic blood collection and flow cytometric analysis of human CD45. When mice showed signs of leukemia development, they were randomized and injected intraperitoneally (i.p.) three times, 8 h apart, with either DBZ (5 mg/kg) or DMSO (vehicle). Each experimental group consisted of at least *n* = 3 mice. After this treatment, mice were sacrificed, and T-leukemia cells were isolated from fully infiltrated spleens to obtain RNA and protein lysates.

To evaluate the in vivo effects of miR-22-3p overexpression, MOLT4 and Jurkat-E6 cells that were previously engineered to express the luciferase reporter gene (FUW-Luc-mCherry-puro or MIGR1-mCherry-Luc2) were transduced with pLenti-III-GFP pre-hsa-miR-22-3p or pLenti-III-GFP empty vector control.

For in vivo experiments, 5 × 10^6^ miR-22-3p-overexpressing or control MOLT4 cells expressing luciferase were i.v. injected into 6–8-week-old female NSG mice. After a 7-day window for tumor engraftment, we evaluated disease progression by bioimaging using the IVIS Spectrum in vivo imaging system (Xenogen Corporation, Caliper Life Sciences, Waltham, MA, USA). Tumor bioluminescence was quantified by integrating the total photonic flux (photons/s, p/s) through a region encircling each mouse as determined by the Living Image software package (Xenogen Corporation). In order to evaluate tumor infiltration, all mice were sacrificed at the same time point, and flow cytometry analysis of CD45-positive cells was used to monitor tumor load in peripheral blood, spleen, and liver tissues. These experiments were authorized by the Italian Ministry of Health (authorization n° 618/2016-PR released the 17th of June 2016).

### 2.4. Statistical Analysis

The results are expressed as mean value ± standard deviation (SD). Statistical testing was performed using Student’s t-test (two-tailed, unpaired). For in vivo experiments, survival curves were estimated using the Kaplan–Meier method, and the difference between them was evaluated using the log-rank test (GraphPad Prism, GraphPad Software, San Diego, CA, USA)). Statistically significant differences are defined as * *p* < 0.05, ** *p* < 0.01, *** *p* < 0.001.

## 3. Results

### 3.1. Identification of Differentially Regulated miRNAs following NOTCH1 Inhibition

In order to identify the miRNAs significantly regulated upon NOTCH1 inhibition in T-ALL, we generated a mouse model of NOTCH1-induced leukemia by retroviral-mediated overexpression of a constitutively active oncogenic mutant form of the NOTCH1 receptor (HD-ΔPEST) in hematopoietic Lin^−^ progenitors, as previously reported [16,17]. Leukemic cells from these mice were then injected into secondary recipients (*n* = 6), and mice bearing tumors were treated with either vehicle (DMSO, *n* = 3) or a potent GSI (DBZ, *n* = 3) [17]. Differential gene expression analysis showed extensive modulation following NOTCH1 inhibition, with 728 downregulated and 878 upregulated genes (FDR < 1%). Moreover, GSEA confirmed the efficacy of anti-NOTCH1 DBZ treatment, as the gene sets related to *NOTCH1* and *MYC* were significantly downregulated after NOTCH1 inhibition (Table S1). 

**Figure 1:**
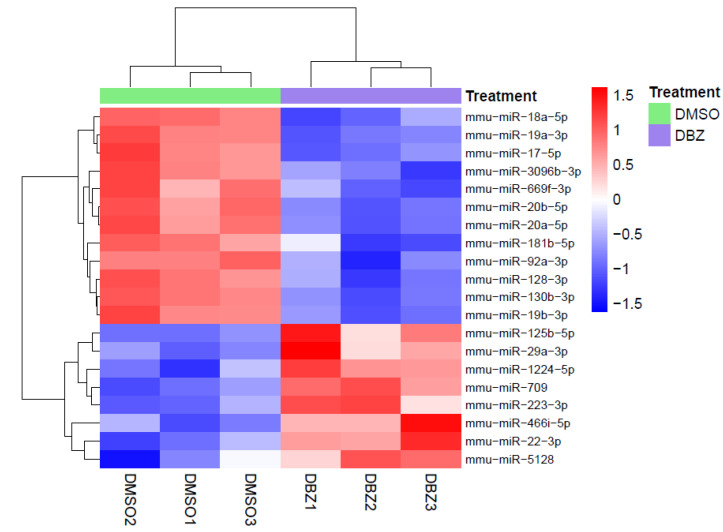
Heat map of significantly regulated miRNAs following NOTCH1 inhibition in vivo. MiRNA expression profiling was performed in three biological replicates of an HD-ΔPEST-*NOTCH1* T-cell tumor treated in vivo with vehicle (DMSO) or with 5 mg/kg of dibenzazepine (DBZ). The heat map shows the significantly differentially expressed miRNAs (|FC| > 1.3; FDR < 0.05). A hierarchical clustering was performed using the Euclidean distance and the complete linkage method on both samples (columns) and miRNAs (rows). The unsupervised analysis clearly separated two clusters representing downregulated miRNAs (top) and upregulated miRNAs (down) in DBZ-treated vs. control.

In parallel, bioinformatic analysis of miRNA expression data from the same HD-ΔPEST-*NOTCH1* tumors under the two experimental conditions revealed 20 differentially regulated miRNAs, with absolute FC > 1.3 and FDR < 5% (Table S2). Specifically, 12 miRNAs were downregulated and 8 were upregulated in DBZ- vs. DMSO-treated samples (Figure 1).

At the top of the list of miRNAs that are downregulated via NOTCH1 following DBZ treatment, we found several components of the miR-17-92 cluster [13], which was previously reported to be highly expressed in T-ALL samples and to cooperate with NOTCH1 at different levels. On the other side, among the eight upregulated miRNAs upon DBZ treatment, we expected to find putative tumor suppressor miRNAs that are usually downregulated in NOTCH1-induced T-ALLs. Interestingly, among them, we found miR-709 and miR-29, which have already been described as tumor suppressors in T-ALL [22,23], and miR-22-3p, which has a known tumor suppressor function in different tumor contexts but has not yet been investigated in NOTCH1-induced leukemia.

### 3.2. miR-22-3p Is Consistently Upregulated Following NOTCH1 Inhibition in Both Murine and Human T-ALL Cells

Defining the role of upregulated miRNAs upon anti-NOTCH1 GSI treatment is challenging and highly intriguing. Among them, there could be potential tumor suppressors that NOTCH1 needs to dampen to exert its oncogenic function. Notably, four miRNAs were conserved between mice and humans: miR-22-3p, miR-223-3p, miR-1224-5p, and miR-29a-3p. We focused on miR-22-3p, which was particularly novel in our setting and produced the most promising and consistent results. Indeed, miR-223-3p has been extensively studied in the context of NOTCH1 inhibition and T-ALL [14,15]. In regard to the two other conserved miRNAs, miR-1224-5p was not expressed in human T-ALL cells (data not shown), and miR-29-3p showed inconsistent results among T-ALL cell lines, exhibiting both upregulation and downregulation upon DBZ treatment without a specific trend (Figure S1).

**Figure 2:**
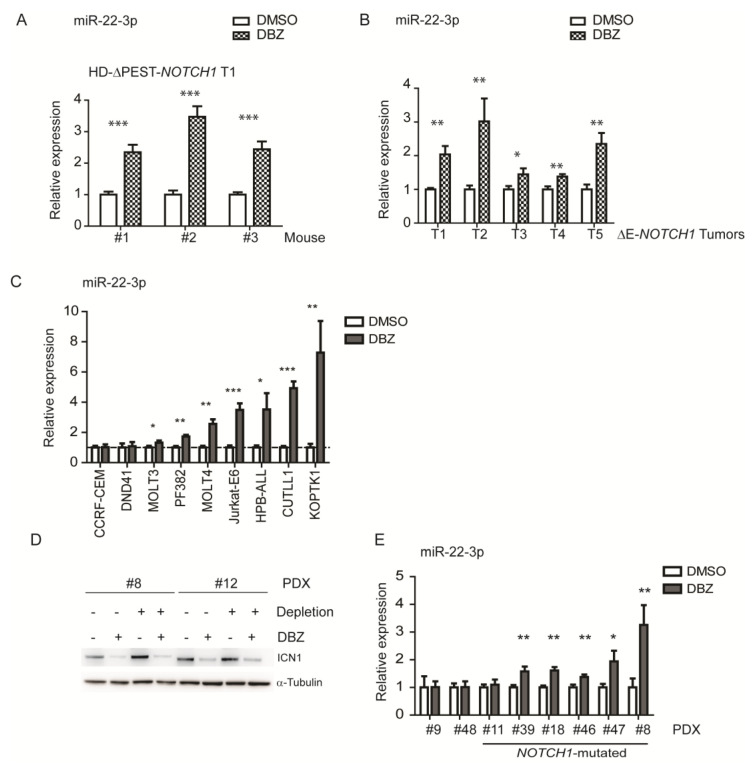
Anti-NOTCH1 treatment in murine and human T-ALL cells results in miR-22-3p upregulation. (**A**,**B**) qPCR analysis of miR-22-3p expression upon DBZ treatment in murine models of NOTCH1-induced T-ALLs: (**A**) leukemic cells obtained from three mice transplanted with the HD-∆PEST-*NOTCH1*-tumor-1 (T1), treated in vivo with DMSO or DBZ, were analyzed; (**B**) five independent ∆E-*NOTCH1*-tumors (T1-5), treated in vivo with DMSO or DBZ, were analyzed. (**C**) qPCR analysis of miR-22-3p expression upon DBZ treatment of T-ALL cell lines (treated in vitro with DBZ for 72 h). (**D**) Western blot analysis of intracellular cleaved NOTCH1 (ICN1) in PDX samples treated in vivo with vehicle only (DMSO) or with 5 mg/kg DBZ, both pre- and post-depletion. α-Tubulin was used as loading control. (**E**) The depleted PDX samples were analyzed by qPCR for miR-22-3p expression. Data are represented as mean ± SD. Assays were performed in triplicate (* *p* < 0.05, ** *p* < 0.01, and *** *p* < 0.001).

Importantly, the upregulation of miR-22-3p following treatment with DBZ was confirmed in murine NOTCH1-induced tumors (HD-∆PEST- and ∆E-*NOTCH1*, Figure 2A,B). Inhibition of the NOTCH1 pathway in ΔE-*NOTCH1* T-leukemia samples was confirmed by Western blot, showing that in DBZ- vs. DMSO-treated cells, there was a strong downregulation in ICN1 protein levels, with *Dtx1*, a direct target of NOTCH1, also significantly decreased (Figure S2). Moreover, shifting to the human setting, we found that miR-22-3p was significantly upregulated following NOTCH1 inhibition in seven out of nine cell lines (Figure 2C). We thus extended the analysis to T-ALL patient-derived xenograft (PDX) samples, previously characterized for their *NOTCH1* and *FBXW7* mutational status (Table S3). To this end, we expanded selected samples in NSG mice, and when mice showed signs of leukemia, we treated them three times, 8 h apart, with either DBZ (5 mg/kg) or DMSO (vehicle). T-cell suspensions obtained from the spleens of sick mice were systematically depleted from murine cells using magnetic beads. In these tumors, ICN1 was strongly downregulated upon DBZ treatment, confirming the efficacy of NOTCH1 inhibition (Figure 2D). miRNA analysis was thus restricted to only the human component, which was enriched from 85% to >95%. Notably, we found that miR-22-3p was significantly upregulated upon GSI treatment in PDX samples bearing *NOTCH1* mutations. PDX #11 was the sole exception because miR-22-3p expression did not change after DBZ treatment, despite being mutated in *NOTCH1* (HD). Interestingly, PDX #9 and #48, which are wild type for *NOTCH1* and *FBXW7* status, did not modulate miR-22-3p upon DBZ treatment.

### 3.3. MiR-22-3p Is Downregulated in T-ALL and Inhibits Cell Growth In Vivo

**Figure 3:**
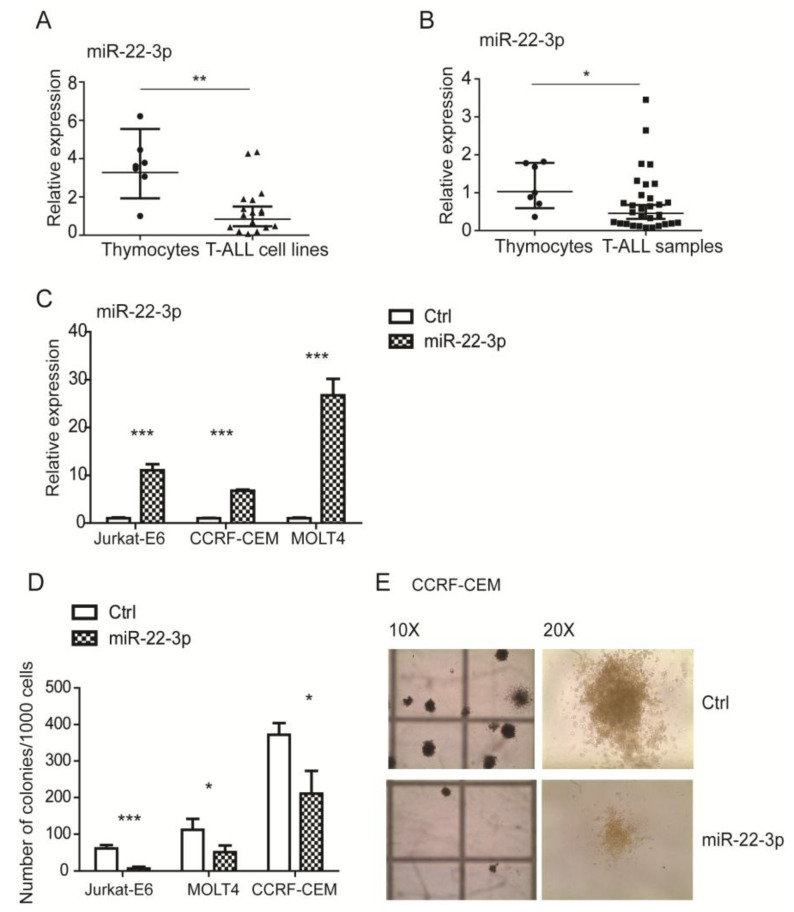
miR-22-3p is downregulated in T-ALL cells and negatively affects colony formation. (**A**,**B**) qPCR analysis of miR-22-3p expression in T-ALL cell lines (*n* = 17) and primary T-ALL samples (*n* = 30) compared with normal thymocytes (*n* = 7). (**C**) qPCR analysis of miR-22-3p overexpression in Jurkat-E6, CCRF-CEM, and MOLT4 cell lines. (**D**,**E**) T-cell lines showing the effects of miR-22-3p overexpression in colony-forming unit (CFU) assays using methylcellulose-based medium (* *p* < 0.05, ** *p* < 0.01, and *** *p* < 0.001). Representative images of CCRF-CEM colonies using an inverted microscope at different magnifications (10× and 20×). Data are represented as mean ± SD. Assays were performed in triplicate. (* *p* < 0.05, ** *p* < 0.01, and *** *p* < 0.001.).

To further investigate the role of miR-22-3p, we analyzed the expression of miR-22-3p in human T-ALL samples, both cell lines and primary samples, with respect to normal immature T cells (normal thymocytes). As expected, miR-22-3p expression was significantly higher in normal human thymocytes (*n* = 7) compared with T-ALL cell lines (*n* = 17, *p* < 0.01; Figure 3A). Importantly, primary T-ALL samples (*n* = 30) also showed reduced expression compared with normal thymocytes (*n* = 7, *p* < 0.05; Figure 3B). In order to investigate the functional effects of miR-22-3p in T-ALL, three cell lines (Jurkat-E6, CCRF-CEM, and MOLT4) were transduced with the pLenti-pre-hsa-miR-22-3p-overexpressing vector. All of them significantly overexpressed miR-22-3p with respect to empty vector controls, as shown by qPCR (*p* < 0.001; Figure 3C). Increased miR-22-3p expression did not affect cell viability under standard or stress culture conditions, such as low-serum medium (data not shown). However, we found that miR-22-3p overexpression significantly affected growth under stress conditions, as revealed through measures such as colony formation in soft agar assays for all three analyzed T-ALL cell lines (*p* < 0.001, *p* < 0.05, and *p* < 0.05 in Jurkat-E6, MOLT4, and CCRF-CEM cells, respectively; Figure 3D). Representative images of control or miR-22-3p-expressing CCRF-CEM cells are shown in Figure 3E.

**Figure 4:**
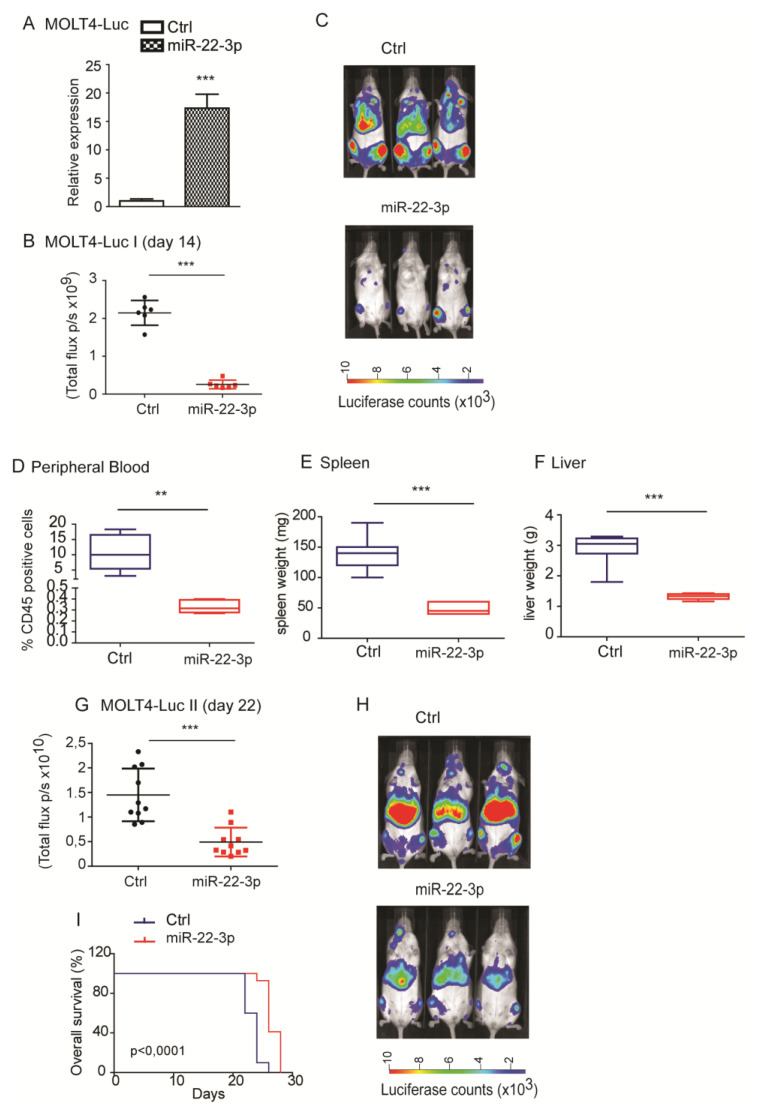
miR-22-3p overexpression in T-ALL cells delays tumor growth in vivo**.** (**A**) MOLT4 cells expressing luciferase were engineered to stably express miR-22-3p or empty vector (ctrl). (**B**,**C**) miR-22-3p or empty vector control MOLT4 cells were injected in vivo in NOD.Cg-*Prkdc^scid^ Il2rg^tm1Wjl^*/SzJ (NSG) mice (*n* = 6). Bioluminescence quantification and representative images of NSG mice at 14 days from injection (*** *p* < 0.001). (**D**–**F**) Flow cytometry analysis of human CD45 expression in the peripheral blood, in the spleen, and in the liver at day 21 after injection (*n* = 6 Ctrl vs. *n* = 6 miR-22-3p overexpressing group; ** *p* < 0.01 and *** *p* < 0.001). (**G**–**I**) Bioluminescence quantification and representative images of NSG mice at day 22 after injection (*n* = 10 Ctrl vs. *n* = 10 miR-22-3p-overexpressing group). Overall survival is shown in (**I**) (*** *p* < 0.001).

Since in vitro miR-22-3p overexpression exerted a significant inhibitory effect on colony-forming assays, in which it strongly reduced the number of colonies, we hypothesized that miR-22-3p overexpression could influence the in vivo growth of T-ALL cells. Notably, luciferase-positive MOLT4 cells (Fuw-Luc-Cherry) that were engineered to overexpress miR-22-3p, as shown in Figure 4A, showed significant in vivo growth delay, as assessed by bioluminescence at day 14 after injection (Figure 4B,C; *n* = 6). At day 21 post-injection, most of the mice in the control group had lost weight and were sick and less active, and all mice were sacrificed to evaluate tumor load. Importantly, infiltration of human T-ALL cells in the peripheral blood, as assessed by flow cytometry analysis of human CD45-positive cells, was significantly reduced in mice injected with MOLT4 cells overexpressing miR-22-3p with respect to those injected with MOLT4 control cells (** *p* < 0.01; Figure 4D). In accordance with this observation, the spleens and liver weights of mice injected with miR-22-3p-overexpressing MOLT4 were significantly lower relative to those of control mice (*** *p* < 0.001; Figure 4E,F). Finally, we evaluated whether miR-22-3p overexpression could affect overall survival. We thus established two groups of mice by injecting MOLT4 cells that express miR-22-3p (miR-22-3p group, *n* = 10) or empty vector (Ctrl group, *n* = 10). At 22 days after injection, we again observed a significant reduction in the bioluminescence signal in mice overexpressing miR-22-3p (*p* < 0.01) relative to that in control mice (Figure 4G,H), similar to the results of the previous experiment. Importantly, Kaplan–Meier curves showed that the overexpression of miR-22-3p was associated with significantly increased survival (*p* < 0.001; Figure 4I). Similar results were also obtained in Jurkat-E6 T-ALL cells, which showed more moderate miR-22-3p overexpression (Figure S3).

In order to dissect the role of miR-22-3p in T-ALL cells, transcriptome analysis of miR-22-3p-overexpressing MOLT4 cells versus empty vector-transduced cells was performed using Clariom S assays (Affymetrix). The results show that miR-22-3p overexpression induced only weak modulation at the transcriptional level, and none of the genes were significantly differentially expressed. A functional enrichment analysis was thus performed using GSEA to investigate possible coordinate deregulation of gene sets related to the molecular function (MF) or biological process (BP) gene ontologies. Interestingly, a gene set related to “alpha-beta T-cell proliferation” in the BP ontology was the only one found to be significantly enriched in terms of downregulation. The genes contributing to the negative enrichment of this gene set are reported in Table S4.

**Figure 5:**
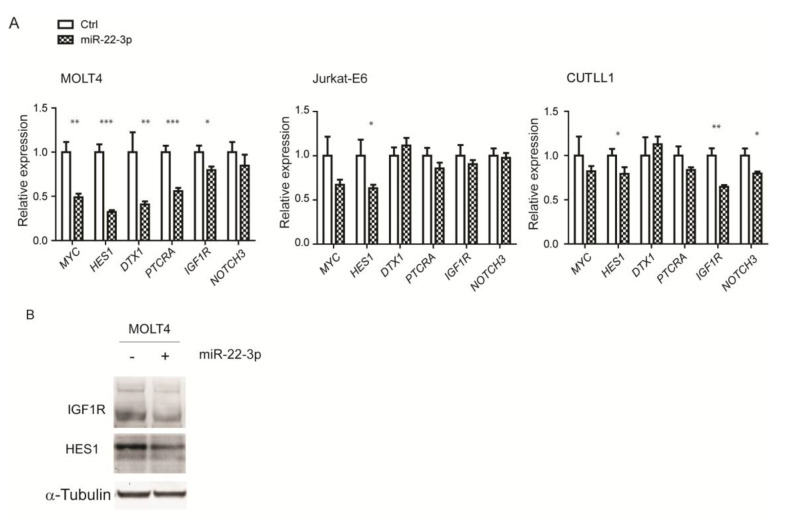
miR-22-3p overexpression in T-ALL cells leads to the downregulation of multiple NOTCH1 targets. (**A**) qPCR analysis of human *MYC, HES1, DTX1, PTCRA IGF1R,* and *NOTCH3* in MOLT4, Jurkat-E6, and CUTLL1 cells engineered to stably express miR-22-3p or empty vector (Ctrl) (* *p* < 0.05, ** *p* < 0.01, and *** *p* < 0.001). (**B**) Western blot analysis of HES1 and IGF1R in MOLT4 cells overexpressing miR-22-3p. α-Tubulin was used as loading control. Data are represented as mean ± SD.

Considering the fact that miRNAs may induce moderate effects on gene expression and that miR-22-3p was identified in the context of NOTCH1 repression, we extended our analysis to specific NOTCH1 targets involved in growth and survival, such as *MYC, HES1, DTX1, PTCRA, IGF1R,* and *NOTCH3*. qPCR analysis in MOLT4, CUTLL1, and Jurkat-E6 cells overexpressing miR-22-3p (~15-, ~25-, and ~10-fold, respectively) showed significant downregulation of multiple targets, particularly *MYC*, *HES1*, and *DTX1* (Figure 5A). Importantly, *HES1*, a critical repressor downstream of NOTCH1, was downregulated upon miR-22-3p overexpression in all three T-ALL cell lines analyzed. To further validate this regulation, we performed Western blot analysis for HES1 and IGF1R, two critical effectors downstream of NOTCH1 signaling, in MOLT4 cells (Figure 5B). Western blot quantification showed downregulation of HES1 and IGF1R proteins (~20% reduction) in MOLT4 cells overexpressing miR-22-3p relative to their levels in control cells.

Overall, these data indicate that miR-22-3p overexpression significantly reduces tumor progression and increases overall survival in vivo, probably through its effects on NOTCH1 oncogenic targets.

## 4. Discussion

T-ALL is an aggressive hematologic tumor that accounts for 10–15% of pediatric and 25% of adult ALL cases. The introduction of intensive combination chemotherapy protocols has led to significant improvements in survival; however, these aggressive regimens are non-specific and very often associated with acute toxicity. The discovery that more than 60% of T-ALL patients harbor mutations in the *NOTCH1* gene has generated new perspectives for the treatment of T-ALL through the use of GSIs or small peptides/antibodies that block NOTCH1 signaling and its regulators. Among the different modulators of NOTCH1 signaling, miRNAs have been shown to play an important role in tumor progression, suggesting the possibility of combining NOTCH1 inhibition with miRNA-based therapy. In this study, we undertook an in vivo approach to identify critical miRNAs that are regulated in the context of NOTCH1 inhibition and that may exert an important function in concert with NOTCH1 in established tumors. We thus used murine NOTCH1-induced T-cell tumors treated in vivo with a highly active GSI to generate paired miRNA and gene expression profiles. Gene expression analysis found numerous genes that were significantly down- and upregulated upon DBZ treatment, in line with previously described results [17]. Importantly, GSEA showed that the gene sets related to NOTCH signaling and MYC targets were significantly downregulated following NOTCH1 inhibition, confirming the efficacy of our experimental model [7,8].

In accordance with previous studies, we identified several components of the miR-17-92 cluster at the top of the list of NOTCH1 downregulated miRNAs, thus reinforcing the relevance of this cluster in the context of NOTCH1-induced T-ALL [11,13]. In fact, dual translocations that simultaneously affect the 17-92 cluster where miR-19 is located and NOTCH1 highlight the oncogenic importance of this interaction in T-ALL [11]. Notably, from our analysis, miR-19a was the most significantly downregulated miRNA upon NOTCH1 inhibition, further supporting its crucial role as an oncomiR in NOTCH1-driven T-ALL. Among the upregulated miRNAs was miR-709, which was previously reported as a tumor suppressor miRNA in the context of NOTCH1-induced leukemia [12]. According to this study, miR-709 represses crucial oncogenes such as Myc, Akt, and Ras-GRF1, playing an important role in murine leukemia initiation and maintenance. Within the miRNAs upregulated upon NOTCH1 inhibition, we also found two conserved miRNAs previously described in the context of T-ALL [23]: miR-29a-3p and miR-223-3p [22,23]. In particular, miR-29 has been reported to function as a tumor suppressor in a NOTCH1-driven T-ALL model and found to be significantly downregulated in T-ALL samples relative to normal thymocytes through the regulation of key genes involved in epigenetic regulatory mechanisms [24,25]. According to our data, in the context of NOTCH1 inhibition, miR-29a-3p showed inconsistent results among human T-ALL cells, exhibiting both upregulation and downregulation without a specific trend. miR-223-3p has been described as an oncomiR in T-ALL because it was found to be highly expressed in T-ALL samples and to cooperate with other miRNAs in regulating key tumor suppressors implicated in T-ALL [13,14]. In our dataset, miR-223-3p was upregulated upon NOTCH1 inhibition, apparently in contrast to its reported oncogenic function. However, our data are in line with another study in which miR-223-3p was found to be upregulated upon NOTCH1 inhibition, implying a complex function for miR-223-3p in T-ALL that may depend on the cell context [15].

Within the miRNAs upregulated upon NOTCH1 inhibition, we also found significant upregulation of miR-22-3p, which has been previously reported as a putative tumor suppressor in acute myeloid leukemia (AML) but never explored in T-ALL, especially downstream of NOTCH1 [22]. We confirmed miR-22-3p modulation upon NOTCH1 inhibition, not only in different NOTCH1-induced tumors, but also in human specimens, such as T-ALL cell lines and PDX samples.

In the last couple of years, miR-22-3p has emerged as a crucial regulator of neoplastic progression. Initially, it was shown that miR-22-3p-overexpressing transgenic mice develop myelodysplastic syndrome, which subsequently progresses to AML [26]. In contrast to these results, Jiang X et al. demonstrated that the forced expression of miR-22-3p significantly suppressed in vitro AML cell viability and growth and substantially inhibited in vivo leukemia development and maintenance [27]. According to these results, miR-22-3p targeted multiple oncogenes, including CRTC1, FLT3, and MYCBP, and thus repressed the CREB and MYC pathways. In line with these data, another study showed that miR-22-3p is a tumor suppressor in AML induced by PU.1, an important transcription factor of monocyte/macrophage differentiation, and reintroduction of miR-22-3p in AML blasts reversed the blocking of differentiation and the inhibition of cell growth [27].

Our data showed that miR-22-3p was repressed in human T-ALL cell lines and PDX samples relative to normal thymocytes. Interestingly, Ghisi M. and collaborators identified miR-22-3p amongst the miRNAs upregulated during normal T-cell development, indicating a different mechanism of regulation of miR-22-3p during the maturation of T cells [28].

Importantly, as previously shown in AML, we demonstrated that overexpression of miR-22-3p in T-ALL cell lines carrying constitutively active NOTCH1 inhibited single-cell growth in soft agar, affecting both the number and size of the colonies. Moreover, miR-22-3p significantly impaired tumor growth in vivo when overexpressed in human T-ALL cells, reducing the percentage of tumor cells in the blood and the size and weight of the spleen and liver.

The striking effects observed in MOLT4 cells overexpressing miR-22-3p can, in part, be explained by the effects of miR-22-3p overexpression on NOTCH1 target genes. Using qPCR, we found that crucial targets downstream of NOTCH1 were significantly downregulated upon miR-22-3p overexpression. For instance, HES1, a known repressor downstream of NOTCH1 that is involved in the survival and growth of T-ALL cells [29], was downregulated in all three analyzed cell lines, and in MOLT4 cells, the corresponding decrease could also be detected at the protein level. Similar results were also obtained in MOLT4 cells for IGF1R, an important regulator of growth and leukemic stem cell activity [6]. It is plausible that these regulations may affect T-ALL cell growth in vivo, where the tumor microenvironment, in concert with NOTCH1 signaling, is a critical factor for tumor establishment and growth.

Overall, these data demonstrate that high miR-22-3p levels can impact tumor progression, possibly affecting the leukemic stem cell compartment in supportive niches and leading to delayed tumor growth.

## Supplementary Materials

The following are available online at https://www.mdpi.com/2073-4409/9/7/1726/s1, Figure S1: Effect of NOTCH1 inhibition on miR-29a-3p expression in human T-ALL cell lines, Figure S2: Effect of NOTCH1 inhibition on ICN1 and Dtx1 expression, Figure S3: miR-22-3p overexpression in Jurkat-E6 cells delays tumor growth in vivo, Table S1: Hallmark gene sets deregulated following GSI treatment in NOTCH1-induced T-ALL in vivo, Table S2: Differentially expressed miRNAs in DBZ- vs DMSO-treated samples, Table S3: NOTCH1 and FBXW7 mutational status in T-ALL PDX samples, Table S4: Genes contributing to the negative enrichment of the ‘GO_ALPHA_BETA_T_CELL_PROLIFERATION’ gene set in MOLT4 overexpressing miR-22-3p.
