# Supplementary material for: miR-22-3p Negatively Affects Tumor Progression in T-Cell Acute Lymphoblastic Leukemia
Source: Cells. 2020 Jul 18;9(7):1726. doi: 10.3390/cells9071726 (PMC7408026; doi:10.3390/cells9071726)
Supplement: Supplementary file 1 [file cells-09-01726-s001.pdf]

Supplementary Materials

RNA extraction, reverse transcription, and qPCR

cDNA was synthesized from 0.5 to 1 μg of total RNA using the Super Script First-Strand Synthesis System (Life Technologies, Carlsbad, CA, USA). Reverse transcription was followed by qPCR reactions, which were performed using the SensiMix SYBR Hi-ROX Kit (Bioline Meridian Bioscience, London, UK) and ABI Prism 7900 Sequence Detection System (Applied Biosystems, Foster City, CA, USA). Quantification of relative gene expression was determined using the ΔΔCt method by normalizing with respect to the geometric mean of two reference genes, *β2M* and *RPL19*. Human-specific primers for *HES1*, *DTX1*, *PTCRA*, and *NOTCH3* have been previously described [1]. Other primer sequences used in the qPCR are reported below.

**Gene**		**Sequence**
**murine *Dtx1***	Forward	5'-AGCTGGTGCCCTACATCATC-3'
**murine *Dtx1***	Reverse	5'-GATGGAGATGTCCATGTCGT-3'
**murine *β2m***	Forward	5'-GTATGCTATCCAGAAAACCC-3'
**murine *β2m***	Reverse	5'-CTGAAGGACATATCTGACATC-3'
**human *β2M***	Forward	5'-AAGGACTCGTCTTTCTATCTC-3'
**human *β2M***	Reverse	5'-GATCCCACTTAACTATCTTGG-3'
**human *RPL19***	Forward	5'-ACGAAAGGGTATGCTCAG-3'
**human *RPL19***	Reverse	5'-CAACCAGACCTTCTTTTTCC-3'
**human *MYC***	Forward	5'-TGAGGAGGAACAAGAAGATG-3'
**human *MYC***	Reverse	5'-ATCCAGACTCTGACCTTTTG-3'
**human *IGF1R***	Forward	5'-AAAGACAAATCCCATCAG-3'
**human *IGF1R***	Reverse	5'-TGCAGGAAATTCTCAAAGAC-3'

On the other hand, to investigate miRNA expression levels, reverse transcription (RT) of RNA into cDNA was performed using miRCURY LNA Universal RT miRNA PCR (Exiqon, Qiagen, Hilden, Germany). Relative miRNA expression was quantified using the ΔΔCt method. Three small non-coding RNAs, used either alone or in combination, were used as references for normalization: two control primer sets (U6 snRNA and SNORD48) and miR-25-3p.

In order to choose a stable endogenous reference miRNA that could also be used in human samples, mouse miRNA microarray data corresponding to 12 samples, 6 HD-ΔPEST-*NOTCH1* (3 DBZ treated and 3 DMSO treated), and 6 ΔE-*NOTCH1* (3 DBZ treated and 3 DMSO treated) were considered. To obtain a more robust result, data were normalized all together using two different methods: (1) RMA normalization, starting from the foreground signal; (2) quantile normalization, starting from the BG subtracted signal produced by Feature Extraction software together with the half option. miRNAs were then ranked according to the variability of their $\log_2$-expression across the 12 samples, leading to two different lists, i.e., one for each normalization method. Among the most stable miRNAs in both lists, we found miR-25-3p, which was chosen as an endogenous reference miRNA. Notably, miR-25-3p emerged as one of the best endogenous reference miRNAs for the normalization of qPCR data in T-ALL [2] in a systematic analysis of miRNA stability performed across a variety of T-lineage samples and T-ALL cell lines.

Plasmids and constructs

pLenti-III-RFP and pLenti-III-GFP (m001, Applied Biological Materials) constructs were used as negative controls or with the sequence of pre-hsa-miR-22-3p (mh12804, Applied Biological Materials) to overexpress the mature miRNA.
The MigR1 vector was kindly provided by Prof. Warren Pear (University of Pennsylvania, Philadelphia, U.S.A) [3]. The Migr1-mCherry-Luc2 vector was obtained by subcloning the fusion protein between the red mCherry fluorescent protein and the luciferase Luc2 protein into ClaI and NcoI restriction sites present in the MigR1 vector as previously described [4]. Migr1 vectors expressing ΔE- and HD-ΔPEST-*NOTCH1* mutant alleles were kindly provided by Prof. Raphael Kopan (Washington University, St. Louis, U.S.A.) and Prof. J. Aster (Brigham and Women's Hospital, Harvard Medical School, Boston, U.S.A.), respectively [5] [6]. The lentiviral vector expressing luciferase FUW-Luc-mCherry-puro was a kind gift from A.L. Kung (Pediatric Department, Columbia University Medical Center, New York, U.S.A.) [7].

Retrovirus and lentivirus production

The production of retroviral and lentiviral particles was executed in human embryonic kidney (HEK) 293T cells. These cells were plated in 10 cm culture dishes (5×10^6 cells) and transfected with a combination of the following vectors: (i) packaging plasmids (retroviral or lentiviral; 2.7 µg) containing *gag*, *pol*, and *rev* genes; (ii) plasmid encoding the envelope gene (*Vesicular stomatitis* virus G glycoprotein; VSV-G; 300 ng); (iii) retroviral (MigRI-based) or lentiviral (pLenti-III-RFP- or pLenti-III-GFP-based) transfer plasmid (3 µg). Forty-eight hours after transfection, the viral supernatants were collected and filtered. Infection of T-ALL cells or Lin^- progenitor cells was performed by spinoculation: cells were resuspended in medium containing virus and hexadimethrine bromide (Polibrene, Sigma Aldrich, St. Louis, MO, USA), and 2×10^6 cells per well were distributed in 24-well plates. Plates were then centrifuged at 2200 rpm for 90 minutes at room temperature and subsequently incubated overnight at 37 °C. Seventy-two hours post-infection, cells were harvested and injected into mice or subjected to puromycin selection (1 µg/mL; Sigma) for 3–5 days or sorted using MoFlo Astrios, a cell sorter from Beckman Coulter (Brea, CA, USA).

Western blotting

Whole-cell lysates were obtained using RIPA buffer (20 mM Tris-HCl (pH 7.5), 15 mM NaCl, 1 mM Na2EDTA, 1 mM EGTA, 1% NP-40, 1% sodium deoxycholate, 2.5 mM sodium pyrophosphate, 1 mM beta-glycerophosphate, 1 mM Na_3VO_4, 1 µg/mL leupeptin) supplemented with phosphatase (50 mM NaF, 1 mM Na_3VO_4) and protease inhibitors (Sigma Aldrich) for 30 minutes on ice. Lysates were then centrifuged at 13,000 rpm for 30 minutes at 4 °C, and supernatants were collected and stored at −80 °C until use. Samples were quantified with the Micro BCA™ Protein Assay Kit (Thermo Scientific, Waltham, MA, USA) according to the manufacturer's instructions. For Western blotting, approximately 20 µg of proteins were separated on 4%–12% gradient NuPAGE® Bis-Tris polyacrylamide or 3%–8% gradient NuPAGE® Tris-Acetate SDS-PAGE gels (Life Technologies) and were then transferred onto nitrocellulose membranes (Amersham Protran, Sigma Aldrich). Membranes were blocked in Dulbecco's phosphate-buffered saline–0.1% Tween-20 (PBS-T) containing 5% nonfat milk and then incubated overnight at 4 °C with primary antibodies. The following primary antibodies were used: anti-β-Actin, anti-cleaved NOTCH1 (Val1744), and anti-HES1 from Cell Signaling Technologies (Denvers, MA, USA); anti-α-Tubulin and anti-IGF1R from Santa Cruz Biotechnology (Dallas, TX, USA). The next morning, membranes were washed three times with PBS-T for 10 minutes each before incubation for 1 hour with the appropriate secondary antibodies conjugated with horseradish peroxidase (HRP). After incubation with the secondary antibody, three washes of 10 minutes each were performed before image acquisition. For immunodetection, nitrocellulose membranes were incubated with chemiluminescence reagent, obtained by mixing equal volumes of Western Lightning® Plus ECL Enhanced Luminol Reagent Plus and Western Lightning® Plus ECL Oxidizing Reagent Plus

(PerkinElmer, Waltham, MA, USA). Images were acquired with BioRad ChemiDoc XRS (Bio-Rad, Hercules, CA, USA) and analyzed with Quantity One® 1-D (Bio-Rad) analysis software.

Cell viability assays

Cell viability analysis was performed using the bioluminescent method Vialight plus (Lonza Group, Basel, Switzerland) after 72 h. The principle of this assay is based on the bioluminescent detection of cellular ATP as a measure of cell viability. This test is based on the quantification of light emission produced by the oxidative reaction of the luciferase enzyme with the luciferin substrate, which requires ATP. Briefly, 100 µL of each P24 well was transferred in triplicate to a 96-well plate and incubated in the dark for 5 minutes at room temperature. Mammalian cell lysis solution (50 µL) was then added followed by incubated for 15 minutes at room temperature. Subsequently, 100 µL of ATPlite substrate was added to each well, and after 2 minutes of incubation, the luminescence signal was detected with PerkinElmer's Victor plate reader.

Clonogenic assay

Colony-forming unit (CFU) assays were performed by plating in 35 × 10 mm Petri dishes with the equivalent of 1000 T-ALL cells overexpressing miR-22 or empty vector control in 1 mL of methylcellulose medium. This medium was composed of 40% MethoCult H4100 (STEMCELL Technologies, Vancouver, Canada) containing 2.6% methylcellulose in Iscove's modified Dulbecco's medium (IMDM), 20% fetal bovine serum, and 40% RPMI 1640 medium complemented with 1% UltraGlutamine, 1% Na-pyruvate, 1% HEPES, 100 U/mL penicillin G, and 100 µg/mL streptomycin (Lonza). Cultures were then incubated at 37 °C in a humidified atmosphere of 5% CO2 in air. Triplicate dishes were prepared for each condition. After 7, 14, and 21 days, colonies were counted using an inverted microscope (Leica, Wetzlar, Germany) and a 60 mm gridded scoring dish.

Mouse Cell Depletion

To enrich the human cells upon xenotransplantation, we used the Mouse Cell Depletion Kit (Miltenyi Biotec, Bergisch Gladbach, Germany). Briefly, cells isolated from infiltrated spleens were resuspended in a specific buffer (PBS, 0.5% bovine serum albumin (BSA), pH 7.2) and added to the Mouse Cell Depletion Cocktail containing monoclonal antibodies conjugated with MicroBeads, which magnetically label mouse cells. After incubation at 4 °C for 15 minutes, the cell suspension was loaded onto a MACS Column that was placed in the magnetic field of a MACS Separator, and the column was then washed two times with the specific buffer. Thus, the magnetically labeled mouse cells were retained within the column, while the unlabeled cells flowed through, allowing the enriched human tumor cells to be collected.

Flow cytometric analysis

To evaluate the level of infiltration of the different mouse tissues by human T-ALL cells, we performed flow cytometry. Briefly, cell suspensions were stained with a specific mouse anti-human CD45 (BD) allophycocyanin (APC)-conjugated antibody according to the manufacturer's instructions. Samples were collected on a FACSCalibur (BD Biosciences, Franklin Lakes, NJ, USA) flow cytometer using Cell Quest software (BD Biosciences).

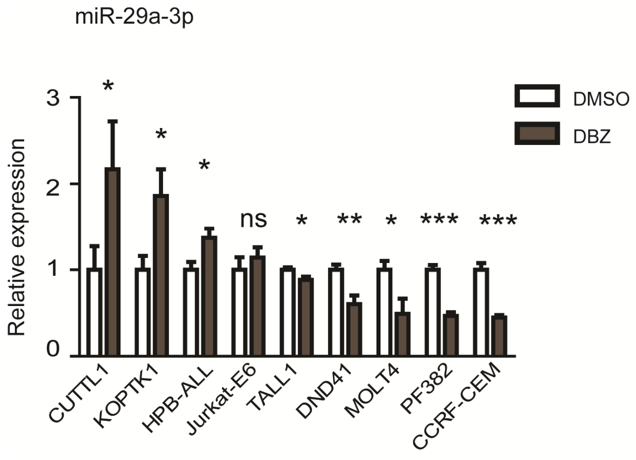

Figure S1. Effect of NOTCH1 inhibition on miR-29a-3p expression in human T-ALL cell lines. qPCR analysis of miR-29a-3p expression upon DBZ treatment in human T-ALL cell lines.

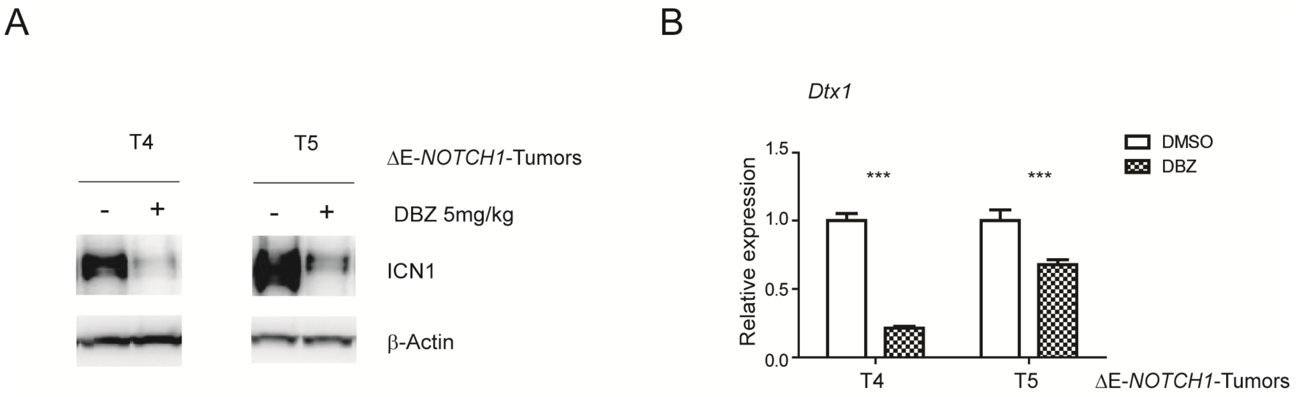

Figure S2. Effect of NOTCH1 inhibition on ICN1 and *Dtx1* expression. (A) Western blot analysis of ICN1 in 2 representative ΔE-*NOTCH1*-Tumors (T4 and T5) treated *in vivo* with DBZ. β-Actin was used as loading control. (B) qPCR analysis of *Deltex* expression upon DBZ treatment in ΔE-*NOTCH1*-Tumors (T4 and T5); data are represented as mean ± SD.

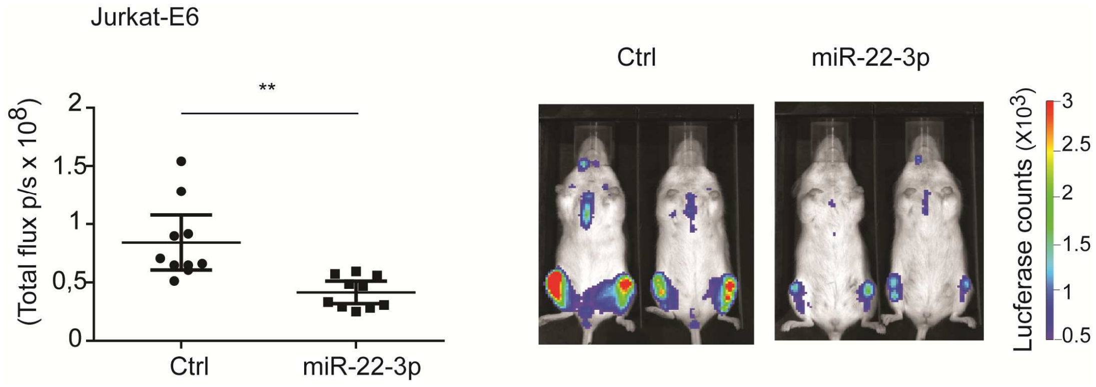

Figure S3. miR-22-3p overexpression in Jurkat-E6 cells delays tumor growth *in vivo*. Bioluminescence quantification and representative images of NSG mice at 14 days after the injection (**P < 0.01).

Table S1. Hallmark gene sets deregulated following GSI treatment in NOTCH1-induced T-ALL *in vivo*. Gene expression profiling using SurePrint G3 mouse GE 8X60K Agilent Microarray was performed using three biological replicates of the same HD-ΔPEST-*NOTCH1* T-cell tumors that were treated *in vivo* with vehicle only (DMSO) or with 5 mg/kg of a potent GSI (DBZ) three times every 8 hours.

**Downregulated Gene Set**	**Size**	**ES**	**NES**	**FWER p-value**
HALLMARK_MYC_TARGETS_V1	184	−0.55	−2.56	0
HALLMARK_MYC_TARGETS_V2	57	−0.61	−2.34	0
HALLMARK_E2F_TARGETS	186	−0.39	−1.83	0.007
HALLMARK_NOTCH_SIGNALING	31	−0.53	−1.78	0.011
HALLMARK_G2M_CHECKPOINT	183	−0.37	−1.71	0.019

**Upregulated Gene Set**	**Size**	**ES**	**NES**	**FWER p-value**
HALLMARK_EPITHELIAL_MESENCHYMAL_TRANSITION	190	0.61	2.75	0
HALLMARK_INTERFERON_GAMMA_RESPONSE	176	0.59	2.63	0
HALLMARK_INTERFERON_ALPHA_RESPONSE	83	0.64	2.58	0
HALLMARK_COAGULATION	132	0.55	2.33	0
HALLMARK_ANGIOGENESIS	35	0.68	2.27	0
HALLMARK_HEME_METABOLISM	178	0.49	2.20	0
HALLMARK_COMPLEMENT	185	0.47	2.12	0
HALLMARK_MYOGENESIS	195	0.42	1.89	0.002
HALLMARK_IL6_JAK_STAT3_SIGNALING	83	0.47	1.86	0.006
HALLMARK_APOPTOSIS	154	0.42	1.86	0.006
HALLMARK_INFLAMMATORY_RESPONSE	197	0.40	1.82	0.012
HALLMARK_IL2_STAT5_SIGNALING	190	0.40	1.80	0.014
HALLMARK_P53_PATHWAY	186	0.40	1.78	0.017
HALLMARK_CHOLESTEROL_HOMEOSTASIS	73	0.45	1.78	0.018
HALLMARK_APICAL_JUNCTION	186	0.39	1.76	0.025

Results of gene set enrichment analysis. Size: number of genes in the expression dataset belonging to the pathway; ES: enrichment score; NES: normalized enrichment score; FWER: family-wise error rate.

Table S2. Differentially expressed miRNAs in DBZ- vs. DMSO-treated samples.

**Probe**	**miRNA**	**FC**	**p-value**	**FDR p-value**
A_54_P1431	mmu-miR-19a-3p	−1.745	2.00E-05	0.002
A_54_P1266	mmu-miR-130b-3p	−1.671	3.00E-05	0.002
A_54_P3922	mmu-miR-20b-5p	−2.007	4.00E-05	0.002
A_54_P1305	mmu-miR-20a-5p	−1.896	5.00E-05	0.002
A_54_P1267	mmu-miR-19b-3p	−2.089	5.00E-05	0.002
A_54_P2476	mmu-miR-18a-5p	−1.603	8.00E-05	0.002
A_54_P2098	mmu-miR-709	1.415	1.60E-04	0.002
A_54_P00004585	mmu-miR-17-5p	−1.386	1.70E-04	0.002
A_54_P00002291	mmu-miR-128-3p	−1.835	1.80E-04	0.002
A_54_P00005485	mmu-miR-3096b-3p	−1.502	3.00E-04	0.004
A_54_P2496	mmu-miR-92a-3p	−1.703	3.30E-04	0.004
A_54_P00004988	mmu-miR-669f-3p	−1.337	0.0010	0.009
A_54_P4402	mmu-miR-1224-5p	1.427	0.0011	0.009
A_54_P1309	mmu-miR-22-3p	1.356	0.0015	0.011
A_54_P2629	mmu-miR-223-3p	1.470	0.0019	0.013
A_54_P2651	mmu-miR-181b-5p	−1.529	0.0024	0.014
A_54_P2282	mmu-miR-125b-5p	1.324	0.0025	0.014
A_54_P00005052	mmu-miR-466i-5p	2.302	0.0030	0.015
A_54_P2488	mmu-miR-29a-3p	1.383	0.0054	0.024
A_54_P00005602	mmu-miR-5128	1.320	0.0109	0.042

FC: fold change; FDR: false discovery rate.

Table S3. *NOTCH1* and *FBXW7* mutational status in T-ALL PDX samples [1,8,9].

PDX	*NOTCH1* status	*FBXW7* status
#8	HD+PEST mut.	wt
#9	wt	wt
#11	HD mut	wt
#18	JME	wt
#39	HD mut.	wt
#46	HD mut.	mut.
#47	HD mut.	mut.
#48	wt	wt

HD, heterodimerization (domain); PEST (domain): proline (P), glutamic acid (E), serine (S), and threonine (T) rich domain; JME (alleles), juxtamembrane expansion (JME) alleles [10].

Table S4. Genes contributing to the negative enrichment of the 'GO_ALPHA_BETA_T_CELL_PROLIFERATION' gene set in MOLT4 cells overexpressing miR-22-3p. Functional enrichment analysis was performed using GSEA to identify coordinate deregulations of GO gene sets due to miR-22-3p overexpression. A single gene set 'GO_ALPHA_BETA_T_CELL_PROLIFERATION' was found to be significantly downregulated (FWER p-value = 0.017) in MOLT4 cells overexpressing miR-22-3p. Genes are ordered according to decreasing rank in the gene list. No gene set was found to be significantly upregulated.

**Gene Symbol**	**Description**	**Rank in Gene List**	**Running ES**
CD28	CD28 molecule	19,200	0.00
VSIR	V-set immunoregulatory receptor	19,075	−0.15
PTPRC	protein tyrosine phosphatase receptor type C	19,044	−0.22
TNFSF4	TNF superfamily member 4	19,036	−0.30
HLA-E	major histocompatibility complex, class I, E	18,905	−0.36
CD274	CD274 molecule	18,438	−0.40
ARG2	arginase 2	18,430	−0.45
IL12B	interleukin 12B	18,273	−0.49
TNFRSF14	TNF receptor superfamily member 14	17,779	−0.51
DOCK2	dedicator of cytokinesis 2	17,775	−0.54
IRF1	interferon regulatory factor 1	16,772	−0.53
RASAL3	RAS protein activator like 3	16,292	−0.53
ITCH	itchy E3 ubiquitin protein ligase	16,291	−0.55
BLM	BLM RecQ like helicase	16,245	−0.58
XCL1	X-C motif chemokine ligand 1	16,189	−0.60
TGFBR2	transforming growth factor beta receptor 2	16,162	−0.62

References

1. Agnusdei, V.; Minuzzo, S.; Frasson, C.; Grassi, A.; Axelrod, F.; Satyal, S.; Gurney, A.; Hoey, T.; Seganfreddo, E.; Basso, G., et al. Therapeutic antibody targeting of Notch1 in T-acute lymphoblastic leukemia xenografts. *Leukemia* **2014**, *28*, 278-288, doi:10.1038/leu.2013.183.
2. Drobna, M.; Szarzynska-Zawadzka, B.; Daca-Roszak, P.; Kosmalska, M.; Jaksik, R.; Witt, M.; Dawidowska, M. Identification of Endogenous Control miRNAs for RT-qPCR in T-Cell Acute Lymphoblastic Leukemia. *International journal of molecular sciences* **2018**, *19*, doi:10.3390/ijms19102858.
3. Pear, W.S.; Miller, J.P.; Xu, L.; Pui, J.C.; Soffer, B.; Quackenbush, R.C.; Pendergast, A.M.; Bronson, R.; Aster, J.C.; Scott, M.L., et al. Efficient and rapid induction of a chronic myelogenous leukemia-like myeloproliferative disease in mice receiving P210 bcr/abl-transduced bone marrow. *Blood* **1998**, *92*, 3780-3792.
4. Piovan, E.; Yu, J.; Tosello, V.; Herranz, D.; Ambesi-Impiombato, A.; Da Silva, A.C.; Sanchez-Martin, M.; Perez-Garcia, A.; Rigo, I.; Castillo, M., et al. Direct reversal of glucocorticoid resistance by AKT inhibition in acute lymphoblastic leukemia. *Cancer cell* **2013**, *24*, 766-776, doi:10.1016/j.ccr.2013.10.022.
5. Schroeter, E.H.; Kisslinger, J.A.; Kopan, R. Notch-1 signalling requires ligand-induced proteolytic release of intracellular domain. *Nature* **1998**, *393*, 382-386, doi:10.1038/30756.
6. Chiang, M.Y.; Xu, L.; Shestova, O.; Histen, G.; L'Heureux, S.; Romany, C.; Childs, M.E.; Gimotty, P.A.; Aster, J.C.; Pear, W.S. Leukemia-associated NOTCH1 alleles are weak tumor initiators but accelerate K-ras-initiated leukemia. *The Journal of clinical investigation* **2008**, *118*, 3181-3194, doi:10.1172/JCI35090.
7. Kimbrel, E.A.; Davis, T.N.; Bradner, J.E.; Kung, A.L. In vivo pharmacodynamic imaging of proteasome inhibition. *Molecular imaging* **2009**, *8*, 140-147.
8. Pinazza, M.; Borga, C.; Agnusdei, V.; Minuzzo, S.; Fossati, G.; Paganin, M.; Michielotto, B.; De Paoli, A.; Basso, G.; Amadori, A., et al. An immediate transcriptional signature associated with response to the histone deacetylase inhibitor Givinostat in T acute lymphoblastic leukemia xenografts. *Cell death & disease* **2016**, *6*, e2047, doi:10.1038/cddis.2015.394.
9. Bordin, F.; Piovan, E.; Masiero, E.; Ambesi-Impiombato, A.; Minuzzo, S.; Bertorelle, R.; Sacchetto, V.; Pilotto, G.; Basso, G.; Zanovello, P., et al. WT1 loss attenuates the TP53-induced DNA damage response in T-cell acute lymphoblastic leukemia. *Haematologica* **2018**, *103*, 266-277, doi:10.3324/haematol.2017.170431.
10. Sulis, M.L.; Williams, O.; Palomero, T.; Tosello, V.; Pallikuppam, S.; Real, P.J.; Barnes, K.; Zuurbier, L.; Meijerink, J.P.; Ferrando, A.A. NOTCH1 extracellular juxtamembrane expansion mutations in T-ALL. *Blood* **2008**, *112*, 733-740, doi:10.1182/blood-2007-12-130096.
